# Low Frequency Repetitive Transcranial Magnetic Stimulation to Improve Motor Function and Grip Force of Upper Limbs of Patients With Hemiplegia

**DOI:** 10.5812/ircmj.13579

**Published:** 2014-08-05

**Authors:** Poopak Motamed Vaziri, Farid Bahrpeyma, Mohammad Firoozabadi, Bijan Forough, Boshra Hatef, Rahman Sheikhhoseini, Aryan Shamili

**Affiliations:** 1 Faculty of Medical Sciences, Tarbiat Modares University, Tehran, IR Iran; 2 Department of Medical Physics, Tarbiat Modares University, Tehran, IR Iran; 3 School of Medicine, Tehran University of Medical Sciences, Tehran, IR Iran; 4 Neuroscience Research Center, Baghiyatallah University on Medical Sciences, Tehran, IR Iran; 5 Department of Physical Education and Sport Sciences, University of Tehran, Tehran, IR Iran; 6 Neuroscience Research Center, Tabriz University of Medical Sciences, Tabriz, IR Iran

**Keywords:** Stroke, Transcranial Magnetic Stimulation, Rehabilitation, Motor Skill

## Abstract

**Background:**

Stroke is the most common and debilitating neurological disorder among adults, and is a sudden onset of neurological signs caused by brain blood vessels impairments.

**Objectives:**

Some new therapeutic methods focus on the use of magnetic stimulation to produce therapeutic effects by inducing the currents. The aim of this study is to determine the effects of rTMS plus routine rehabilitation on hand grip and wrist motor functions in patients with hemiplegia, and compare with pure routine rehabilitation programs.

**Patients and Methods:**

In this study, 12 patients with hemiplegia were randomly divided in two groups. Control group, received the rehabilitation program with placebo magnetic stimulation, and the experimental group, received magnetic stimulation with routine rehabilitation program for 10 sessions for three times per week. Pre and post evaluations of treatment performed using Barthel and Fugl-Meyer indices and dynamometers.

**Results:**

In the control group, Barthel and Fugl-Meyer indices showed significant improvement (P = 0.01, P = 0.00), while in the experimental group, significant improvement in Barthel and Fugl-Meyer indices and dynamometers has been observed (P = 0.01, P = 0.00, P = 0.007).

**Conclusions:**

rTMS can improve hand muscle force and functions of patients with chronic hemiplegia, while conventional treatment is not effective.

## 1. Background

Stroke is the most common and debilitating neurological disorder among adults, and is a sudden onset of neurological signs caused by brain blood vessels impairments. Stroke is the third leading cause of death in the world after cardiovascular diseases and cancers. Stroke is the main cause of more than 10-12% of all deaths and more than 50% of survivors suffer from long term disabilities. Generally, stroke is divided in two main types: Ischemic and hemorrhagic strokes. About 70% of strokes are ischemic type, 20% are hemorrhagic and 10% are idiopathic (1, 2).

Brain stroke is considered as the upper motor neuron diseases (2). Patient's main problem is uncoordinated movement patterns associated with abnormal postural tones. Hemiplegia is the classic symptom of CVA (Cerebrovascular Accident) (3). According to previous study, patients that CVA resulted in functional and neurological disorders, rehabilitation was an effective procedure and could improve the functional abilities. It has been identified that age has no effect on the rehabilitation process. Focal neurological deficits resulted from stroke can reflect the injury size and zone and collateral flow rate (3).

Functional movement therapy is developed as a new therapeutic approach in recent years. It is a combination of past approaches and those functional movements used in weight bearing or non-weight bearing patterns to increase joint mobility and to reduce the hyper tonicity in muscles of upper or lower limbs. The principles of this approach is: obtaining full range of motion in all joints by complex functional patterns and joint mobility, eliminating muscle imbalance by stretch and strengthening, and motor control restoration of affected limb (4).

In 1951, Twitchel came up with a theory, which is still remaining as a topic: He stated that after stroke, the involvement of the upper limbs is more than those of lower limb and the improvement in upper limb achieved with more delay and slower recovery. Post stroke weakness in shoulder and time restoration of hand movements are two important characteristics that define the recovery level in upper limbs (5). Repetitive transcranial magnetic stimulation (rTMS) is a novel technique that is widely used for the treatment of depression, mania, schizophrenia, Parkinson, epilepsy and chronic pains. In recent years rTMS is used in post-stroke cares (6). While recent progresses in improving the stroke care have been primarily concentrated on the neuroprotective and neurovascular diseases, tools used to study and alter cortical function have played a significant role in all parts of post stroke care including diagnostic, prognostic, and interventional (7).

Based on the type of stimulation, TMS can affect nervous system in two ways: Single or paired pulse TMS causes neurons to depolarize and discharge an action potential in the brain cortex beneath the stimulated area (8). Long lasting effects of TMS is obtained by repetitive TMS. Depend on the intensity of stimulation, coil orientation and frequency; rTMS can change the excitability of the corticospinal tracts. Although the mechanism is not clear but it is widely believed that rTMS can cause changes in synaptic efficacy akin to long term potentiation (LTP) and long term depression (LTD) (9). Safety, ethical considerations and application guidelines for the use of TMS have been approved by Rossi et al. (10).

Most of the rehabilitation hemiplegic treatments focus on lower extremities and the fact that disabilities in upper limbs are common and persistent, cause a necessity for newer treatments. Different routine rehabilitation techniques are used to improve the function in hemiplegic patients and according to the results, it is recommended that addition of new treatments for chronic stage is necessary because of less helpfulness of traditional approaches. Several studies are performed to assess the effect of rTMS on motor function and grip strength of upper limbs in patients with hemiplegia (11-19). In these studies different frequencies and treatment sessions to only assess the presence of motor function and grip strength, but, according to authors measuring both variables in one study using highly safe frequencies (1 Hz) of rTMS in combination with routine rehabilitation has not been performed till now. In some other studies, only the study group underwent rehabilitation and the patients in the control group did not receive rehabilitation (17, 19-23).

## 2. Objectives

In fact, this study was conducted to determine the effects of rTMS accompanied by routine rehabilitation on hand grip and wrist motor functions in patients with hemiplegia, and to compare with pure routine rehabilitation programs.

## 3. Patients and Methods

### 3.1. Subjects

For this clinical trial study, the study protocol was reviewed and approved by the Ethics Committee of the Tarbiat Modares University, Tehran, Iran (No: 52/84482, Date: 10 January 2011), which was performed in Firoozgar Hospital (General hospital, 450 beds, governmental and referral), Tehran, Iran. According to previous studies (15, 16, 18) to achieve 80% probability (β = 0.20) of detecting and 20% difference (α = 0.05) in improvement among two groups, at least six subjects is necessary for each group. In this clinical trial study, 12 volunteers were selected purposely. They were visited by a physician for the inclusion criteria and then were randomly divided to either the experimental (rTMS plus rehabilitation, age 55.17 ± 5.42 years, height 171.83 ± 0.90 cm, weight 84.50 ± 10.86 kg, BMI 28.53 ± 1.86 and time duration after stroke 24.00 ± 8.29 months) and the control (routine rehabilitation, age 57.00 ± 8.67 years, height 168.50 ± 0.13 cm, weight 75.33 ± 11.14 kg, BMI 26.44 ± 0.97 and time duration after stroke 23.00 ± 8.94 months) groups through a simple randomization selection.

Inclusion criteria included hemiplegia in dominant side after single stroke, middle cerebral artery involvement, spasticity due to stroke, at least two months after stroke, 30 to 65-year-old men and women. The exclusion criteria included stroke due to cardiac embolism, permanent injuries of upper extremities like fractures, neurologic disorders like Parkinsonism, multiple sclerosis and etc, upper extremity mobility restriction due to other reasons, epilepsy or family history of epilepsy, intracranial implantation or clips, pacemaker, lesion in occipital, limbic system and complementary area, incapable to work for four weeks. At first, in a familiarization session subjects have been orally and written informed about the procedure and the aim of the study and signed an informed consent form. Demographic characteristics of every participant (such as: gender, age, weight, height, job, history of stroke, motor disabilities according to the subject opinion, previous physical therapy management, etc.) were collected by an administrated questionnaire.

### 3.2. Data Collection

To assess the motor recovery, balance, sensation, pain and the range of joint motion according to the Brunnstrom Approach, Fugl-Meyer questionnaire was used. The reliability and validity of this scale to assess the upper and lower extremities motor function and as a stroke severity stratification variable among different stroke recovery time points was established previously (24, 25). Recently, Sullivan et al. (26) showed that intra-rater reliability of the expert-base was high in the motor and sensory scores (range, 0.95-1.0). Inter-rater agreement between expert and therapist raters was high for the motor scores (total, 0.98; upper extremity, 0.99; lower extremity, 0.91) and sensory scores (total, 0.93; light touch, 0.87; proprioception, 0.96). Assessment of motor function of upper extremity was performed in 33 tasks and each was scored from zero (complete disability) to two (full, coordinate and normal performance), because Persian translation of this questionnaire was not available, questionnaire administered by interview.

Martin Vigorimeter was used to measure the grip strength. The maximum value in three trials by each patient was recorded and considered as grip strength. Molenaar et al. (27) showed that the intra-class correlation coefficient for the Martin vigorimeter was 0.84 (95% confidence interval, 0.77 to 0.89) for the dominant hand and 0.86 (95% confidence interval, 0.80 to 0.90) for the non-dominant hand. Barthel index is used for the functional assessment of participants. The reliability of Barthel index has been proofed in post CVA patients (28) and its Persian version has excellent reliability and validity (29). The index is an ordinal scale comprising ten activities of daily living. The original BI was scored as five points to give a maximum total score of 100 (30). One day before the training sessions started and a day after the final session, all subjects were tested by same expert physical therapist (P.M) and under the same condition.

### 3.3. Interventions

In the control group, subjects received routine rehabilitation program for upper extremity. After 10 minutes of Faradic electrical stimulation for wrist and finger extensor muscles, patients exercised the functional movements for 30 minutes in the same predetermined program. Rehabilitation program Included: upper extremity functional patterns, elbow, wrist and fingers mobility, gentle stretch of hypertonic muscles, strengthening of weak muscles in weight bearing and non-weight bearing patterns, muscle imbalance elimination, motor control restoration of involved extremities, reduction of muscle stiffness and motion restoration. To eliminate placebo effect of rTMS, the program followed by using 20 minutes of of rTMS system. This procedure performed 10 sessions totally comprised of three 60-minutes sessions per week.

The experimental group underwent a same rehabilitation program followed by real rTMS treatment. According to Takeuchi et al. study: “rTMS system was set at 1 Hz for 20 minutes with 60-80% of motor threshold using continuous current and intensity of 1.5-2 Tesla on coil surface. rTMS was performed with a 100 mm figure-8 coil. The coil was placed tangentially on the contralesional M1 at the optimal site for the first dorsal interosseous (FDI) muscle. The optimal site was defined as the location where stimulation of a slightly supra threshold intensity stimulated the largest MEPs in the FDI. Electromyographic (EMG) activity was recorded from silver-silver-chloride electrodes positioned in a belly tendon montage on the skin overlying the FDI, and the signal was amplified, filtered (50 to 2000 Hz) and digitized at a sampling rate of 5000 Hz for off line analysis” (31). Then the current increased up to the FDI muscle showed a minimal contraction, which was considered as a patient's motor threshold.

### 3.4. Data Analysis

Descriptive statistics were used for subject's demographic characteristics. The one sample Kolmogorov Smirnov test used to check the normal assumption. For pre-test and post-test results, paired samples t-tests were performed to find statistically significant differences between the mean value of variables in the control and experimental groups. Student's t-tests were used to find any statistically significant difference between the mean value of each variable in pre-tests and post-tests results. Confidence interval was considered as95% (P value > 0.05). Data analyzed by SPSS.19 software.

## 4. Results

In order to compare the two groups during the pre-test and post-test times, independent t-test was performed and the results showed that there were no significant differences between the mean values of three variables (Table 1). Mean scores of Fugl-Myere (P < 0.001) and Barthel index (P = 0.01) questionnaires significantly increased in the control group but there was no significant difference between the mean values of grip strength in pre-test and post-test measures (P = 0.108) (Table 1). After treatment by rTMS the mean scores of Fugl-Meyer (P < 0.001) and Barthel index (P = 0.01) questionnaires significantly increased. On the contrary in the control groups, the mean value of grip strength showed statistically significant increase in post-test measures in comparison to the pre-test measures (P = 0.001) (Table 1).

**Table 1.** Comparison Between Variable of Measurement in Both Groups

	Experimental				Control				
	Pre-test	Post-test	P Value 1 ^a,b^	Differences	Pre-test	Post-test	P Value 1	Differences	P Value 2
**Fugl-Meyer index**	19 ± 2.45	26.5 ± 2.88	0.001	-7.5 ± 1.38	17 ± 3.95	23 ± 4.83	0.001 ^a^	-6 ± 0.63	0.133
**Barthel index**	68.33 ± 14.02	78.33 ± 14.02	0.010	-10.00 ± 7.09	73.33 ± 6.06	80 ± 4.48	0.011 ^a^	-6.67 ± 4.08	0.791
**Vigorimeter measures**	6.83 ± 4.88	10.5 ± 4.93	0.001	-3.67 ± 0.84	3.17 ± 2.71	6.00 ± 4.10	0.054	-2.83 ± 3.54	0.197

^a^ Significant Difference was Observed.

^b^ Data are presented as mean ± SD.

## 5. Discussion

**Figure 1.**
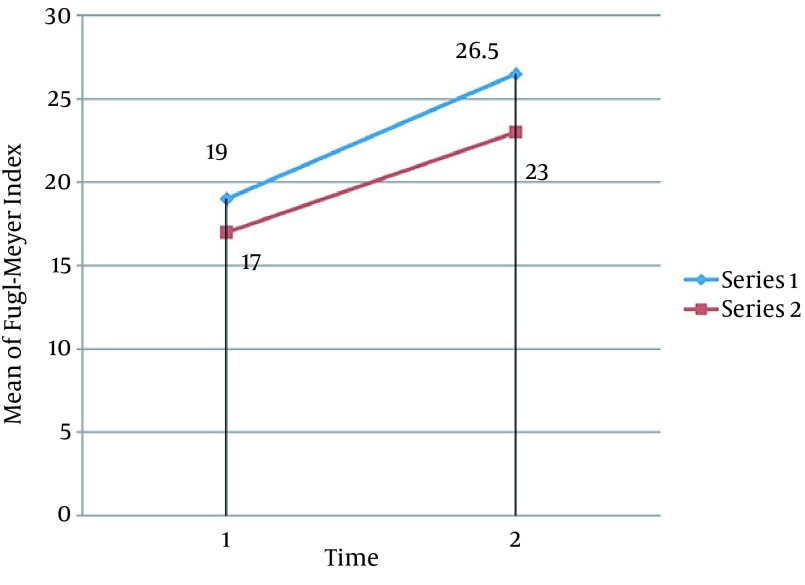
Comparison of Mean of Fugl-Meyer Index Between the Two Groups Mean values of three variables increase in both groups during time but in the experimental group this amount increases with the little bigger slope. (Series 1: Experimental Group, Series 2: Control Group. 1: Pre-test, 2: Post-test)

**Figure 2.**
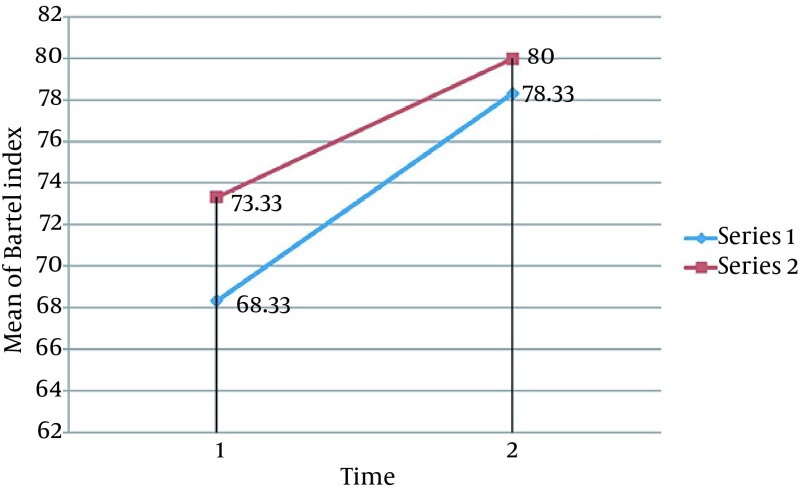
Comparison of Mean of Bartel Index Among the 2 Groups Mean values of three variables increase in both groups during time but in the experimental group this amount increases with the little bigger slope. (Series 1: Experimental Group, Series 2: Control Group. 1: Pre-test, 2: Post-test)

**Figure 3.**
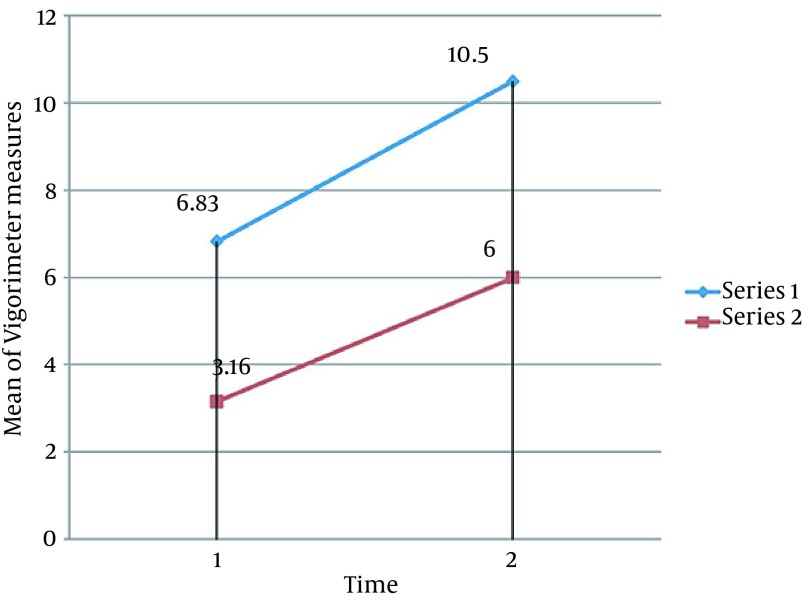
Comparing the Mean of Vigorimeter Measures Comparison Among the 2 Groups Mean values of 3 variables increases in both groups during time but in Experimental Group increase with the little bigger slope. (Series 1: Experimental Group, Series 2: Control Group. 1: Pre-test, 2: Post-test)

No adverse side effects were reported during or after the study. Statistical analysis of Fugl-Meyer, Barthel indices and Martin Vigorimeter measures after 10 sessions showed significant improvements in all variables of both groups, except for the Martin Vigorimeter measures in the control group (P < 0.05). Our results showed that rTMS plus routine rehabilitation in post-stroke patients resulted in a better functional motor function compared to the routine rehabilitation (Figures 1, 2 and 3). Several studies showed that rTMS may be resulted in grip strength in post stroke patients (11-14, 21, 32). Improvement in Barthel indices and grip strength measures in the experimental group is in accordance to that stated by Khedr et al. (33), showing that in 10 consecutive days, an additional rTMS intervention to the normal physical and drug therapies improves immediate clinical outcome in early stroke patients. Improvement in Fugl-Meyer measures in the experimental group is consistent with several studies (15, 16, 18).

Khedr et al. (22) showed that with sham stimulation comparison, 1 Hz over M1 of the unaffected hemisphere, significantly increased the motor cortex excitability of the affected hemisphere and decreased cortical excitability of the unaffected hemisphere. Another possible mechanism of rTMS to achieve motor function recovery may be facilitating practice dependent plasticity and improving the motor regenerating performance in post stroke patients (11). Gershon et al. (34) reported that rTMS can reduce depression, and laboratory studies on rats showed that transcranial magnetic field stimulation induced neurogenesis of the sub ventricular zone (35), these may be two possible mechanisms result in functional improvements in patients.

This study demonstrates the mean of Barthel index and Fugl-Meyer measures improved significantly after routine rehabilitation in the control group, but the improvement of grip strength was not significant. The results are consistent with several studies (36-41) that stating that this improvement is due to the increased range of motion and functional exercises during rehabilitation programs. Some of researchers suggested that to gain more functional recovery in post stroke patients, longer rehabilitation and more intensity of training are needed and patients should be in acute or sub-acute phase (36, 42, 43). Strong point of our study is that we compare rTMS plus routine rehabilitation effects with the routine rehabilitation plus Sham rTMS. It can eliminate the placebo effect of rTMS. The limitation of this study was the limited sample size. It is obvious if more subjects were participated in the study, the differences between the two groups could be detected more clearly. Also to discover the intracranial changes, intracranial assessment equipment must be used in future studies.

The main outcome of this study is that rTMS plus routine rehabilitation can increase grip strength more than routine rehabilitation solely. Because grip strength has been shown to be a predictor of disability and mortality in older adults, remediation of low grip strength should be an important aspect of treatment for individuals with stroke (44). Thus it seems that using rTMS plus routine rehabilitation program for post stroke patients can accelerate restoration of function and decrease disability in shorter time.

## References

[1] Tyson SF, Hanley M, Chillala J, Selley A, Tallis RC (2006). Balance disability after stroke.. Phys Ther..

[2] Tink Martin S,, Kessler M (2006). Neurologic Intervention for Physical Therapist Assistants..

[3] Bobat B (2002). [Adult Hemiplegia Evaluation and Treatment]..

[4] Ryerson S, Levit K (1997). Functional Movement Reeducation: A Contemporary Model for Stroke Rehabilitation..

[5] Delisa JA, Gans BM, Walsh NE (2005). Physical Medicine and Rehabilitation: Principles and Practice..

[6] Machado S, Bittencourt J, Minc D, Portella CE, Velasques B, Cunha M (2008). Therapeutic applications of repetitive transcranial magnetic stimulation in clinical neurorehabilitation.. Funct Neurol..

[7] Dimyan MA, Cohen LG (2010). Contribution of transcranial magnetic stimulation to the understanding of functional recovery mechanisms after stroke.. Neurorehabil Neural Repair..

[8] Theodore WH (2002). Handbook of Transcranial Magnetic Stimulation. Edited by A. Pascual-Leone, N.J. Davey, J. Rothwell, E.M. Wasseran, B.K. Puri, Arnold, London, 2001. pound 110 sterling, ISBN 0340720093.. Epilepsy Behav..

[9] Fitzgerald PB, Fountain S, Daskalakis ZJ (2006). A comprehensive review of the effects of rTMS on motor cortical excitability and inhibition.. Clin Neurophysiol..

[10] Rossi S, Hallett M, Rossini PM, Pascual-Leone A, Safety of TMSCG (2009). Safety, ethical considerations, and application guidelines for the use of transcranial magnetic stimulation in clinical practice and research.. Clin Neurophysiol..

[11] Kim YH, You SH, Ko MH, Park JW, Lee KH, Jang SH (2006). Repetitive transcranial magnetic stimulation-induced corticomotor excitability and associated motor skill acquisition in chronic stroke.. Stroke..

[12] Cogiamanian F, Marceglia S, Ardolino G, Barbieri S, Priori A (2007). Improved isometric force endurance after transcranial direct current stimulation over the human motor cortical areas.. Eur J Neurosci..

[13] Yozbatiran N, Alonso-Alonso M, See J, Demirtas-Tatlidede A, Luu D, Motiwala RR (2009). Safety and behavioral effects of high-frequency repetitive transcranial magnetic stimulation in stroke.. Stroke..

[14] Takeuchi N, Toshima M, Chuma T, Matsuo Y, Ikoma K (2008). Repetitive transcranial magnetic stimulation of the unaffected hemisphere in a patient who was forced to use the affected hand.. Am J Phys Med Rehabil..

[15] Kakuda W, Abo M, Kaito N, Ishikawa A, Taguchi K, Yokoi A (2010). Six-day course of repetitive transcranial magnetic stimulation plus occupational therapy for post-stroke patients with upper limb hemiparesis: a case series study.. Disabil Rehabil..

[16] Kakuda W, Abo M, Kobayashi K, Momosaki R, Yokoi A, Fukuda A (2010). Low-frequency repetitive transcranial magnetic stimulation and intensive occupational therapy for poststroke patients with upper limb hemiparesis: preliminary study of a 15-day protocol.. Int J Rehabil Res..

[17] Theilig S, Podubecka J, Bosl K, Wiederer R, Nowak DA (2011). Functional neuromuscular stimulation to improve severe hand dysfunction after stroke: does inhibitory rTMS enhance therapeutic efficiency?. Exp Neurol..

[18] Kakuda W, Abo M, Kobayashi K, Takagishi T, Momosaki R, Yokoi A (2011). Baseline severity of upper limb hemiparesis influences the outcome of low-frequency rTMS combined with intensive occupational therapy in patients who have had a stroke.. PM R..

[19] Mansur CG, Fregni F, Boggio PS, Riberto M, Gallucci-Neto J, Santos CM (2005). A sham stimulation-controlled trial of rTMS of the unaffected hemisphere in stroke patients.. Neurology..

[20] Boggio PS, Alonso-Alonso M, Mansur CG, Rigonatti SP, Schlaug G, Pascual-Leone A (2006). Hand function improvement with low-frequency repetitive transcranial magnetic stimulation of the unaffected hemisphere in a severe case of stroke.. Am J Phys Med Rehabil..

[21] Takeuchi N, Tada T, Toshima M, Chuma T, Matsuo Y, Ikoma K (2008). Inhibition of the unaffected motor cortex by 1 Hz repetitive transcranical magnetic stimulation enhances motor performance and training effect of the paretic hand in patients with chronic stroke.. J Rehabil Med..

[22] Khedr EM, Abdel-Fadeil MR, Farghali A, Qaid M (2009). Role of 1 and 3 Hz repetitive transcranial magnetic stimulation on motor function recovery after acute ischaemic stroke.. Eur J Neurol..

[23] Khedr EM, Etraby AE, Hemeda M, Nasef AM, Razek AA (2010). Long-term effect of repetitive transcranial magnetic stimulation on motor function recovery after acute ischemic stroke.. Acta Neurol Scand..

[24] Duncan PW, Propst M, Nelson SG (1983). Reliability of the Fugl-Meyer assessment of sensorimotor recovery following cerebrovascular accident.. Phys Ther..

[25] Gladstone DJ, Danells CJ, Black SE (2002). The fugl-meyer assessment of motor recovery after stroke: a critical review of its measurement properties.. Neurorehabil Neural Repair..

[26] Sullivan KJ, Tilson JK, Cen SY, Rose DK, Hershberg J, Correa A (2011). Fugl-Meyer assessment of sensorimotor function after stroke: standardized training procedure for clinical practice and clinical trials.. Stroke..

[27] Molenaar HM, Zuidam JM, Selles RW, Stam HJ, Hovius SE (2008). Age-specific reliability of two grip-strength dynamometers when used by children.. J Bone Joint Surg Am..

[28] Murdock C (1992). A critical evaluation of the Barthel Index: II.. Br J OccupTher..

[29] Oveisgharan S, Shirani S, Ghorbani A, Soltanzade A, Baghaei A, Hosseini S (2006). Barthel index in a Middle-East country: translation, validity and reliability.. Cerebrovasc Dis..

[30] Sainsbury A, Seebass G, Bansal A, Young JB (2005). Reliability of the Barthel Index when used with older people.. Age Ageing..

[31] Takeuchi N, Chuma T, Matsuo Y, Watanabe I, Ikoma K (2005). Repetitive transcranial magnetic stimulation of contralesional primary motor cortex improves hand function after stroke.. Stroke..

[32] Dafotakis M, Grefkes C, Eickhoff SB, Karbe H, Fink GR, Nowak DA (2008). Effects of rTMS on grip force control following subcortical stroke.. Exp Neurol..

[33] Khedr EM, Ahmed MA, Fathy N, Rothwell JC (2005). Therapeutic trial of repetitive transcranial magnetic stimulation after acute ischemic stroke.. Neurology..

[34] Gershon AA, Dannon PN, Grunhaus L (2003). Transcranial Magnetic Stimulation in the Treatment of Depression.. Am J Psychiatry..

[35] Arias-Carrion O, Verdugo-Diaz L, Feria-Velasco A, Millan-Aldaco D, Gutierrez AA, Hernandez-Cruz A (2004). Neurogenesis in the subventricular zone following transcranial magnetic field stimulation and nigrostriatal lesions.. J Neurosci Res..

[36] Thrasher TA, Zivanovic V, McIlroy W, Popovic MR (2008). Rehabilitation of reaching and grasping function in severe hemiplegic patients using functional electrical stimulation therapy.. Neurorehabil Neural Repair..

[37] Mangold S, Schuster C, Keller T, Zimmermann-Schlatter A, Ettlin T (2009). Motor training of upper extremity with functional electrical stimulation in early stroke rehabilitation.. Neurorehabil Neural Repair..

[38] Mandic M, Rancic N (2011). The recovery of motor function in post stroke patients.. Med Arh..

[39] Page SJ, Maslyn S, Hermann VH, Wu A, Dunning K, Levine PG (2009). Activity-based electrical stimulation training in a stroke patient with minimal movement in the paretic upper extremity.. Neurorehabil Neural Repair..

[40] Hsu SS, Hu MH, Wang YH, Yip PK, Chiu JW, Hsieh CL (2010). Dose-response relation between neuromuscular electrical stimulation and upper-extremity function in patients with stroke.. Stroke..

[41] Lin Z, Yan T (2011). Long-term effectiveness of neuromuscular electrical stimulation for promoting motor recovery of the upper extremity after stroke.. J Rehabil Med..

[42] Cooke EV, Mares K, Clark A, Tallis RC, Pomeroy VM (2010). The effects of increased dose of exercise-based therapies to enhance motor recovery after stroke: a systematic review and meta-analysis.. BMC Med..

[43] Plavsic A, Svirtlih L, Stefanovic A, Jovic S, Durovic A, Popovic M (2011). [Effects of functional electrical therapy on upper extremity functional motor recovery in patients after stroke--our experience and future directions].. Med Pregl..

[44] Harris JE, Eng JJ (2010). Strength training improves upper-limb function in individuals with stroke: a meta-analysis.. Stroke..

